# NBR1: The archetypal selective autophagy receptor

**DOI:** 10.1083/jcb.202208092

**Published:** 2022-10-18

**Authors:** Nikoline Lander Rasmussen, Athanasios Kournoutis, Trond Lamark, Terje Johansen

**Affiliations:** 1 Autophagy Research Group, Department of Medical Biology, University of Tromsø-The Arctic University of Norway, Tromsø, Norway

## Abstract

NBR1 was discovered as an autophagy receptor not long after the first described vertebrate autophagy receptor p62/SQSTM1. Since then, p62 has currently been mentioned in >10,000 papers on PubMed, while NBR1 is mentioned in <350 papers. Nonetheless, evolutionary analysis reveals that NBR1, and likely also selective autophagy, was present already in the last eukaryotic common ancestor (LECA), while p62 appears first in the early Metazoan lineage. Furthermore, yeast-selective autophagy receptors Atg19 and Atg34 represent NBR1 homologs. NBR1 is the main autophagy receptor in plants that do not contain p62, while most animal taxa contain both NBR1 and p62. Mechanistic studies are starting to shed light on the collaboration between mammalian NBR1 and p62 in the autophagic degradation of protein aggregates (aggrephagy). Several domains of NBR1 are involved in cargo recognition, and the list of known substrates for NBR1-mediated selective autophagy is increasing. Lastly, roles of NBR1 in human diseases such as proteinopathies and cancer are emerging.

## Teaser

Rasmussen et al. discuss NBR1 as the archetypical selective autophagy receptor, as well as its known cargos, roles in diseases, and collaboration with p62.

## The selective autophagy receptor NBR1

Selective autophagy consists of a set of evolutionarily conserved pathways for targeted lysosomal degradation of macromolecules, protein aggregates, lipid droplets, viral capsids, intracellular pathogens, and organelles. The different pathways of selective autophagy depend on either soluble or membrane-bound selective autophagy receptors (SARs; Lamark and Johansen, 2021). The first SAR discovered, p62/SQSTM1 (sequestosome-1), belongs to a family of soluble SARs including NBR1, CALCOCO1, CALCOCO2 (aka NDP52), TAX1BP1 (aka CALCOCO3), and OPTN (optineurin). This group of SARs, commonly referred to as sequestosome-1 like receptors (SLRs; Deretic, 2012), are typically characterized by the presence of (1) an LC3 interacting region (LIR) motif, (2) homo- or hetero-oligomerization domains, and (3) a C-terminal ubiquitin-binding domain for engaging ubiquitinated substrates (Johansen and Lamark, 2020; Nthiga et al., 2020). The SLR–LIR motifs bind to ATG8 family proteins anchored in the autophagosomal double membrane through a covalent conjugation to phosphatidylethanolamine (Lystad and Simonsen, 2019; Mizushima et al., 2011). Substrates and cargos for selective autophagy are usually labeled with ubiquitin or other “eat me” signals recognized by the ubiquitin-binding domain or other domains found in SLRs. When bound to cargo, some SLRs can themselves initiate autophagosome formation in situ by interacting with components of the core autophagy machinery (Chang et al., 2021; Goodall et al., 2022). Further, SLRs can facilitate the expansion of the autophagosome membrane (phagophore) by multivalent interactions with ATG8 proteins (Johansen and Lamark, 2020). The most studied SLR p62 forms phase-separated bodies in cells that are called p62 bodies (Lamark and Johansen, 2021). The formation of p62 bodies depends on polymerization mediated by the N-terminal Phox/Bem1p (PB1) domain (Lamark et al., 2003; Wilson et al., 2003), with p62 forming helical filaments (Ciuffa et al., 2015; Jakobi et al., 2020), and is induced by binding of p62 to polyubiquitin, causing a phase separation (Sun et al., 2018; Zaffagnini et al., 2018). Phase separation of p62 filaments is also induced by increased p62 expression or by posttranslational modifications increasing the binding of p62 to ubiquitin (Lamark and Johansen, 2021).

**Figure 1.**
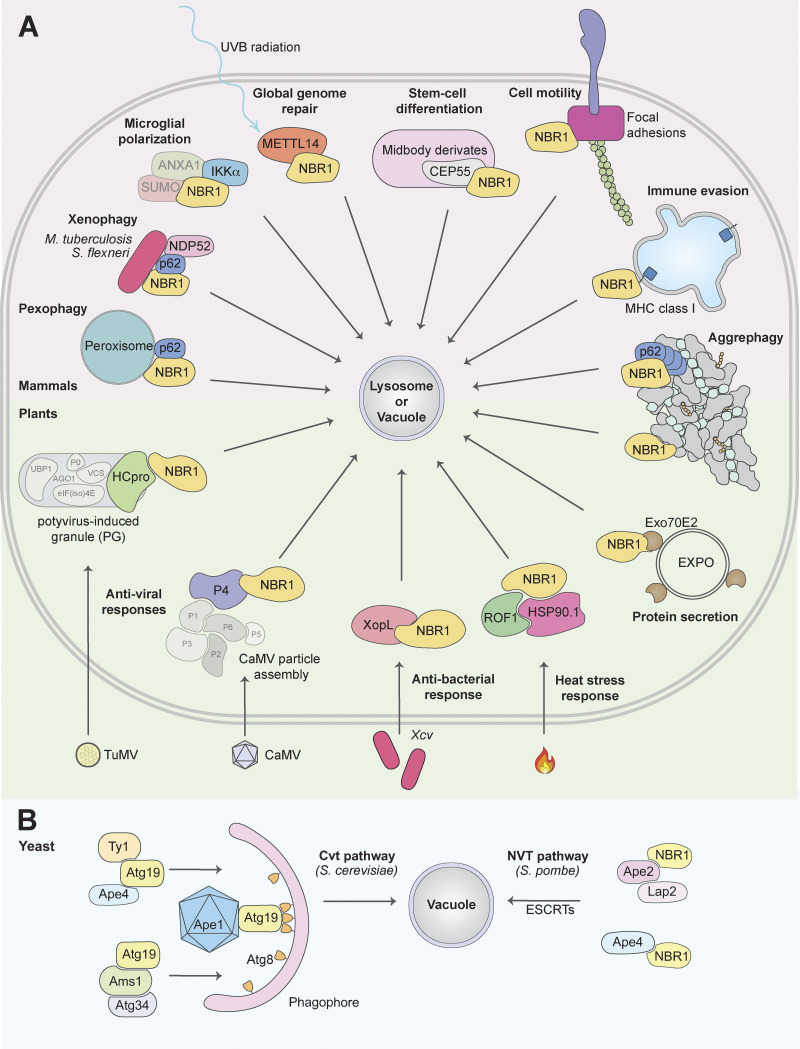
**NBR1 as a selective autophagy receptor in mammals, plants, and yeast.** Summary of identified substrates of NBR1-mediated selective autophagy, indicated by immediate vicinity to NBR1. This does not distinguish direct or indirect interaction. Transparent proteins are part of a complex. **(A)** In mammals, NBR1 has been shown to mediate the degradation of peroxisomes (pexophagy), bacteria (xenophagy), and protein aggregates (aggrephagy) in conjunction with p62 (and NDP52 for xenophagy). Furthermore, NBR1 has been shown to affect several processes through selective autophagic degradation of the following substrates: the proinflammatory kinase IKKα, affects microglial polarization following ischemia (Li et al., 2021); METTL14 (methyltransferase-like 14) upon ultraviolet B radiation, consequently affecting global genome repair (Yang et al., 2021); the midbody protein CEP55 upon stem-cell differentiation (Kuo et al., 2011); turnover of focal adhesions, promoting cell motility; and MHC class I proteins in PDAC cells, promoting immune evasion. In plants, NBR1 regulates several plant stress responses: clearance of aggregates (aggrephagy), restricting TuMV infection by targeting the viral RNA silencing suppressor component HCpro; restricting CaMV infection by targeting the viral particle protein P4; targeting the bacteria effector protein XopL for degradation, restricting Xcv infection; promoting heat stress recovery by targeting ROF1 and HSP90.1; targeting Exo70E2, a marker for the exocyst-positive organelle (EXPO; Ji et al., 2020). **(B)** In *S. cerevisiae*, the NBR1 homolog Atg19 mediates the degradation of Ty1, Ape4, Ape1, and Ams1 through the Cvt pathway. The second NBR1 homolog Atg34, only targets Ams1. In *S. pombe*, the NBR1 homolog targets Ape2, Lap2, and Ape4 to the vacuole. This Nbr1-mediated vacuolar targeting (NVT) pathway is mediated by ESCRTs, not macroautophagy. Only references not cited in the main text are cited here.

NBR1 (neighbor of BRCA1 gene 1) was discovered as a selective autophagy receptor due to its interaction with and similarity in domain organization to p62 and direct binding to ATG8 proteins and ubiquitin (Kirkin et al., 2009; Waters et al., 2009). Mammalian NBR1 acts as a SAR involved in degrading protein aggregates (aggrephagy; Kirkin et al., 2009), peroxisomes (pexophagy; Deosaran et al., 2013), midbody remnants (Isakson et al., 2013; Kuo et al., 2011), focal adhesions (Kenific et al., 2016), and major histocompatibility complex (MHC) class I receptor (Yamamoto et al., 2020). In plants, NBR1 degrades protein aggregates upon heat-, oxidative-, salt-, and drought stress (Zhou et al., 2013; Zhou et al., 2014), viral capsids (Hafren et al., 2017), a viral RNA silencing suppressor (Hafren et al., 2018), and acts in defense against bacterial infections (Leong et al., 2022; Ustun and Hofius, 2018). In fungi, NBR1 homologs transport lysosomal enzymes from the cytoplasm into the vacuole as shown in the fission yeast *Schizosaccharomyces pombe* (Liu et al., 2015; Wang et al., 2021), and they act as a pexophagy receptor in the filamentous fungus *Sordaria macrospora* (Werner et al., 2019). Substrates of NBR1 in mammals, plants, and yeast are summarized in Fig. 1. In this review, we will elaborate on the roles of NBR1 in selective autophagy processes in plants, fungi, and mammals, and its roles in human disease. But first, we will focus on the domain structure and evolution of NBR1.

## Domain structure of NBR1

**Figure 2.**
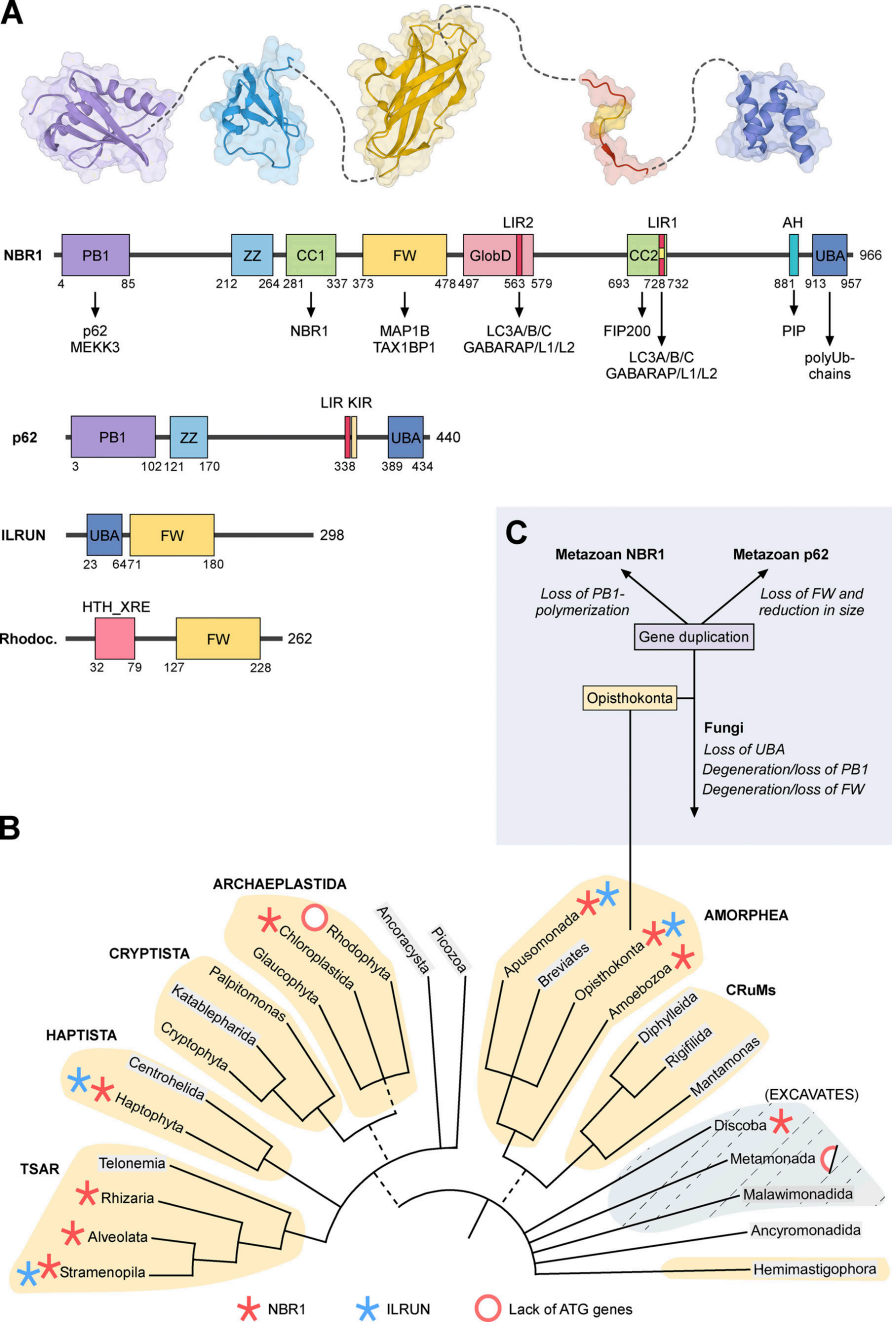
**Domain structure and evolution of NBR1. (A)** Domain architectures of human NBR1, p62, and ILRUN, and HTH-XRE (Helix-turn-helix XRE-family like protein) from *Rhodococcus fascians*. The amino acid positions of the domain borders and the length of the proteins are indicated by numbers below and to the right of the cartoons, respectively. Structures of PB1, ZZ, FW, LIR, and UBA domains are shown above the NBR1 domain architecture. **(B)** Distribution of NBR1 (red asterisk) and ILRUN (blue asterisk) on The New Tree of Eukaryotes. Red ring, or half-red ring, indicates the lack of ATG genes in some clades. The colored groupings are the current “supergroups.” Multifurcations indicate unresolved branching orders among lineages while broken lines represent minor uncertainties about the monophyly of certain groups (Burki et al., 2020). We searched the NCBI Protein Database and the Conserved Domains Database to identify NBR1 and ILRUN homologs (Lu et al., 2020). The phylogenomic distribution was also determined using the SMART database (Letunic et al., 2006). The names of the groupings where sequence data were not available are indicated in gray. **(C)** The Ophistokonta contains both the metazoans and fungi. The gene duplication and divergence of the ancestor NBR1 gene in the early metazoan lineage, and loss of UBA and loss or degeneration of PB1 domains in fungi are indicated.

Vertebrate NBR1 and p62 share an N-terminal PB1 domain, the ZZ zinc finger domain, LIR motif, and C-terminal UBA domains. In addition, NBR1 contains the four tryptophan (FW) domains involved in protein–protein interactions, two coiled-coil (CC) domains, and an amphipathic helix (AH) domain not found in p62 (Deosaran et al., 2013; Mardakheh et al., 2010; Svenning et al., 2011; Fig. 2 A). The PB1 domain is involved in interactions with other PB1 domain-containing proteins (notably p62, see below), while the CC1 domain mediates self-interaction. The UBA domain binds to ubiquitin and ubiquitinated cargo, while the LIR binds to ATG8 family proteins (Johansen and Lamark, 2020). Human NBR1 has two LIR motifs. Both bind ATG8 proteins in vitro, but only LIR1 binds strongly in cell extracts and is required for efficient autophagic degradation of NBR1 (Kirkin et al., 2009).

The FW domain was so named because it contains four highly conserved tryptophan (W) residues (Svenning et al., 2011). It is also referred to as NBR1-like or NBR1 domain (Kraft et al., 2010). Some bacterial proteins also contain FW domains, which precede the eukaryotic NBR1 that first appeared in protists (Marchbank et al., 2012; Svenning et al., 2011; Fig. 2 A). The FW domain is present in only one other eukaryotic protein called ILRUN (inflammation and lipid regulator with UBA-like and NBR1-like domains; Fig. 2 A). The FW domain of human NBR1 binds to microtubule-associated protein MAP1B and TAX1BP1 (Marchbank et al., 2012; Turco et al., 2021). The recent finding that the FW domain of the filamentous fungus *Chaetomium thermophilum* binds specifically to vacuolar α-mannosidase (Ams1) and delivers Ams1 to the vacuole by autophagy in the fission yeast *S. pombe* shows that this domain can be involved in cargo recognition (Zhang et al., 2022a).

The same group previously showed that the *S. pombe* NBR1 homolog uses its ZZ domains to transport aminopeptidases Ape4, Ape2, and Lap2, and Ams1 from the cytosol into the vacuole, analogous to Atg19 acting as a receptor in the biosynthetic cytoplasm to vacuole transfer (Cvt) pathway in *S. cerevisiae* (Liu et al., 2015; Wang et al., 2021; Fig. 1 B). Ams1 and Ape4 bind competitively to ZZ1. Lap2 and Ape2 bind to ZZ2 and ZZ3. Surprisingly, this Nbr1-mediated vacuolar targeting (NVT) pathway in *S. pombe* is not mediated by autophagy components but by endosomal-sorting complexes required for transport (ESCRTs) in a process similar to microautophagy (Hama et al., 2021; Wang et al., 2021). Interestingly, the ZZ domain of mammalian p62, but not that of mammalian NBR1, is also used for cargo recognition by binding to N-arginylated proteins (Cha-Molstad et al., 2017).

The AH domain of mammalian NBR1 was identified as a 22 amino-acid amphipathic α-helical structure based on secondary structure predictions (Mardakheh et al., 2010). It is located adjacent to the UBA domain and was initially called the JUBA domain for juxta-UBA. Since JUBA and UBA are confusingly similar names both verbally and written, we propose to call this domain simply AH for amphipathic α-helix. Circular dichroism spectroscopy showed the AH domain to be an unfolded structure that folds into an α-helix in the presence of membranes containing phosphatidylinositol-phosphates (PIPs; Mardakheh et al., 2010). AH displayed no specificity toward a specific PIP. Both the AH and UBA domains are needed for the co-localization of NBR1 with LAMP2 (late endosomes; Mardakheh et al., 2010) and peroxisomes (Deosaran et al., 2013), suggesting AH is required for membrane localization of NBR1.

## Evolution of NBR1: The archetypal soluble autophagy receptor

Autophagy-related (ATG) genes have experienced expansions and losses during the evolution of different eukaryotic lineages, enabling functional diversification and specialization. Remote homologs of ATG proteins and the evolutionarily conserved protein domains are found in bacteria and archaea. These were likely recruited into the developing autophagy pathway during eukaryogenesis (Zhang et al., 2021). Phylogenetic and biochemical analyses reveal the evolutionary relationship between NBR1 and p62. Using the presence of the FW domain to distinguish between p62 and NBR1 homologs, we found NBR1 orthologs to be distributed throughout the eukaryotic kingdom, while p62 is confined to the metazoans (Svenning et al., 2011; Fig. 2, B and C). Most non-metazoan organisms have only a single NBR1 homolog and no p62 homolog. Metazoans generally contain both NBR1 and p62, but NBR1 has been secondarily lost in some animal lineages including nematodes, insects, and crustaceans. Clearly, NBR1 preceded p62 in evolution, and p62 likely arose through gene duplication of the ancestral NBR1 gene, which happened early in the metazoan lineage (Fig. 2 C). This is illustrated by the fact that the choanoflagellate *Monosiga brevicollis* and the amoeban protist *Capsaspora owczarzaki*, representing the closest living unicellular relatives of metazoans (King et al., 2008; Ruiz-Trillo et al., 2004), have only a single NBR1 homolog and no p62 homolog (Svenning et al., 2011).

Autophagy is a very fundamental pathway appearing at the root of eukaryote evolution and is likely present in the last eukaryotic common ancestor (LECA; Zhang et al., 2021). LECA is defined as the ancestor of all existing eukaryotes, plus extinct post-LECA lineages. LECA likely arose 1.9–1.6 billion years ago, with all the main features of a eukaryotic cell (Spang et al., 2022). With a few exceptions like red algae, microsporidia, and the flagellate intestinal parasite *Giardia lamblia*, ATG genes and autophagy are found throughout eukaryotes (Zhang et al., 2021; Zhang et al., 2022b). Although gene loss and expansions occur in many lineages, the two conjugation systems with ATG8 and ATG12 are conserved in most eukaryotic clades (Zhang et al., 2022b). The origin of selective autophagy likely occurred with the first SAR, and NBR1 is the pioneer soluble SAR. None of the other vertebrate SARs have been found in protists or plants. Defining NBR1 homologs as proteins that as a minimum contain an FW domain and a PB1 or ZZ-type zinc finger domain, we find NBR1 homologs in five of the supergroups of the newly proposed tree of eukaryotes (Burki et al., 2020). Specifically, representatives of the TSAR supergroup (including Stramenopila, Alveolata, and Rhizaria), Haptista (Haptophyta), Archaeplastida (Chloroplastida), Amorphea (including Apusomonada, Amoebozoa, and Ophistokonta), and Discoba all have NBR1 homologs (Fig. 2 B). Opisthokonta includes animals, fungi, and some protist lineages that are most closely related to either animals or fungi. Chloroplastida includes green algae and land plants. All the remaining supergroup taxa mentioned represent protists (Burki et al., 2020). The lack of sequence data for some important species defining a few of the taxa presently precludes an exhaustive analysis. However, the coincident presence of important core ATG proteins makes it tempting to suggest that the ancestor NBR1 may have been present in LECA, representing the first SAR that evolved. Hence, selective autophagy may have originated in the LECA and co-evolved with unselective autophagy.

Apart from NBR1, ILRUN is the only other eukaryotic protein containing an FW domain (Fig. 2 A). It is not clear if there is a functional relationship between NBR1 and ILRUN. Human ILRUN is a 298 amino acid protein (formerly known as C6orf106) containing an N-terminal UBA-like domain (residues 23–64) and a central FW domain (residues 71–180). We traced the homologs of ILRUN protein in the evolution, guided by the eukaryotic tree of life, and found that ILRUN is present in all metazoans including the simplest metazoan *Trichoplax adhaerens* and the closest unicellular relatives to metazoans *Monosiga brevicollis* and *Capsaspora owczarzaki*. ILRUN homologs are also found in the Stramenopila of the TSAR supergroup, Haptophyta of the Haptista supergroup, and Apusomonada, but not the sister group Amoebozoa of the Amorphea supergroup (Fig. 2 B). Intriguingly, distinct from NBR1, ILRUN is not found in plants and fungi. This suggests a secondary loss of ILRUN in these taxa.

## Atg19 and Atg34 are yeast NBR1 homologs

**Figure 3.**
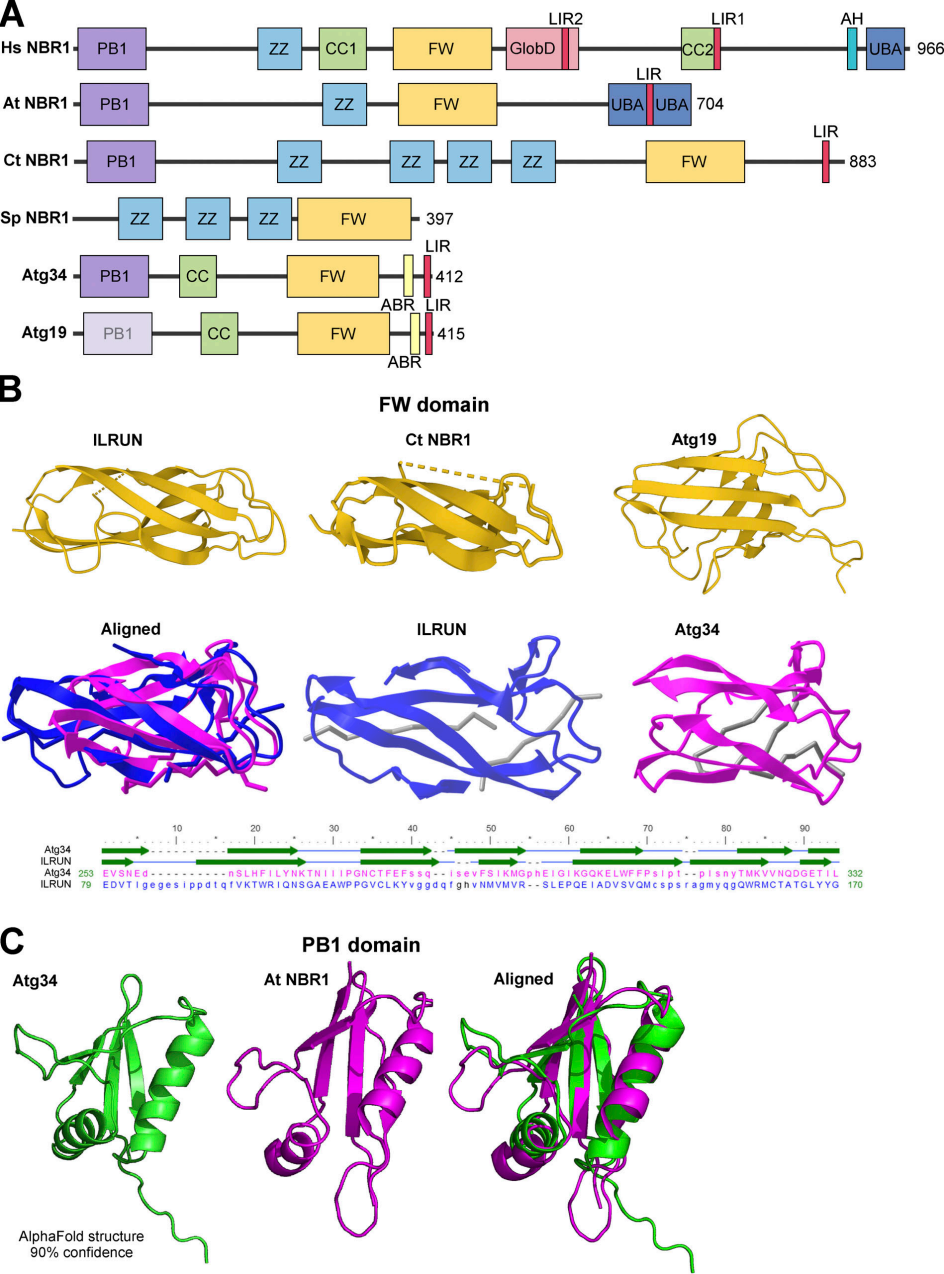
**The yeast Cvt receptor Atg19 and the paralog Atg34 are NBR1 homologs. (A)** Domain architecture of NBR1 homologs from humans, the plant *Arabidopsis thaliana*, the filamentous fungus *C. thermophilum,* fission yeast *S. pombe, S. cerevisiae* Atg19 and Atg34. **(B)** Comparison of FW domain structures between human ILRUN (PDB accession no. 6VHI), *C. thermophilum* (Ct NBR1; PDB accession no. 7VQO), and the ABD structures from yeast Atg19 (PDB accession no. 2KZB) and Atg34 (PDB accession no. 2KZK). Structural alignment of the ILRUN FW domain (blue) to the Atg34 ABD/FW domain (magenta) obtained by a VAST search of the PDB database. A sequence alignment with positions of the structural elements (β strands) is shown below the structures. Despite only 7% sequence identity the alignment gives a root-mean-square deviation of 1.90 Å over a 60 amino acid sequence. **(C)** Atg34 contains a PB1 domain fold. The AlphaFold structure predicted with 90% confidence for the N-terminal domain of Atg34 (green) is a PB1 domain that can be structurally aligned to the solved structure (PDB accession no. 6TGN) of the PB1 domain of *Arabidopsis thaliana* (magenta). The structures were aligned using PyMol.

*S. cerevisiae*, *D. melanogaster*, and *C. elegans* are extremely valuable model organisms. However, due to long divergent evolution with gene duplications and loss they are often the “odd ones out” when it comes to sequence-based evolutionary studies of proteins. A seminal perspective article suggested that Atg19 is the NBR1 homolog in *S. cerevisiae* (Kraft et al., 2010). Acting as a receptor in the Cvt pathway, yeast Atg19 was the first selective autophagy receptor discovered (Leber et al., 2001; Scott et al., 2001). The primary cargo in the Cvt pathway is the precursor form of the vacuolar aminopeptidase 1 (preApe1), which forms a tetrahedral dodecameric structure that is recognized by the CC domain of Atg19. Atg19 recruits Atg8 via its C-terminal LIR (often called Atg8-family interaction motif [AIM] in yeast) and the selective autophagy adapter Atg11. This Cvt complex recruits the core autophagy machinery to initiate membrane formation and expansion to form the Cvt vesicle, a special type of autophagosome only 150 nm in diameter (reviewed in Yamasaki and Noda, 2017). In addition to Ape1, Atg19 also transports the vacuolar aspartyl aminopeptidase Ape4, the vacuolar α-mannosidase Ams1, and even the Ty1 retrotransposon particle to the vacuole. Almost 10 yr after the discovery of Atg19, its paralog Atg34 was discovered (Suzuki et al., 2010). Atg19 and Atg34 have the same domain organization (Fig. 3 A) and show 31% overall sequence identity (49% similarity), but Atg34 can only target Ams1. Hence, Atg34 cannot compensate for Atg19 in the Cvt pathway (Yamasaki and Noda, 2017).

Atg19 has lost the ZZ zinc-finger domain and has no sequence similarity to NBR1 homologs in other phyla. However, Kraft, Peter, and Hofmann noted that Atg19 has the LIR domain and predicted that highly divergent PB1 and NBR1 folds were present in *S. cerevisiae* Atg19, as well as a CC domain (Kraft et al., 2010). Their argument also rested on the evolutionary analysis of NBR1 with emphasis on fungal homologs, where some fungal lineages had retained NBR1 with PB1, ZZ, FW, LIR, and UBA domains although having two or three copies of the ZZ domain. Most fungi lost the UBA domain, and PB1 and FW domains could not be identified by sequence conservation in evolved lineages such as *Saccharomyces*, but a similar fold was predicted, suggesting the presence of PB1-like and FW-like domains in *S. cerevisiae* Atg19 (Kraft et al., 2010; Fig. 3 A). The solution structures of the α-mannosidase binding domains (ABD) of Atg19 and Atg34 are solved (Watanabe et al., 2010). The Atg19 and Atg34 ABD structures are very similar, with a root mean square difference (RMSD) of 2.1 Å for 102 residues, forming an immunoglobulin-like β-sandwich fold with two β-sheets, each with four antiparallel β-strands. The ABD of the NBR1 homolog of the filamentous fungus *C. thermophilum* has an FW domain very similar in structure to those found in human NBR1 and ILRUN (Zhang et al., 2022a). Structural comparison of FW domain structures of NBR1 and ILRUN with the ABD of Atg19 and Atg34 revealed the latter to be FW-like domains (Fig. 3 B). A VAST structural alignment search of the Protein Database with the Atg34 ABD revealed the human ILRUN FW domain as a structural homolog of the ABD. With the ABD of Atg19 and -34 being structurally homologous to FW domains, we asked if the N-terminal regions of these two yeast proteins may be PB1-like domains. Comparing the PB1 domain structure of *Arabidopsis* NBR1 (Jakobi et al., 2020) to AlphaFold structure prediction with 90% confidence of the *S. cerevisiae* Atg34 N-terminal region (Jumper et al., 2021; Varadi et al., 2022) clearly suggest the presence of a PB1 domain in Atg34 (Fig. 3 C). In Atg19 the prediction is very uncertain, but the presence of a PB1-like domain is likely. Structure determinations of the N-terminal regions of Atg19 and Atg34 will give us clear answers. However, taken together, we suggest that Atg19 and Atg34 are clearly NBR1 homologs with PB1/PB1-like, CC, FW, and LIR domains (Fig. 3 A).

## An early metazoan gene duplication created the paralogs p62/SQSTM1 and metazoan NBR1

The split of ancestor NBR1 into the current paralogs p62/SQSTM1 and NBR1 in vertebrates was likely initiated by a gene duplication very early in metazoan evolution (Svenning et al., 2011; Fig. 2 C). Further evolution led to one shortened paralog lacking the CC and FW domains (p62) and one full-length with a modified and monomeric PB1 domain (metazoan NBR1). The gain of an AH domain may have occurred before vertebrates evolved. To understand the functional consequence of the duplication event, it is important to relate it to the role of the PB1 domain in selective autophagy. PB1 is a ubiquitin-like domain that engages in homomeric or heteromeric PB1–PB1 interactions. The interaction involves two individual and oppositely charged binding surfaces. A negatively charged binding surface in one PB1 domain binds to a positively charged binding surface in the other (Jakobi et al., 2020; Lamark et al., 2003; Wilson et al., 2003). Individual PB1 domains may contain one or both binding surfaces. PB1 domains with both binding surfaces can result in homomeric polymerization of the PB1-containing protein, as seen for mammalian p62. Cryo-EM analyses demonstrated that the PB1 domain of p62 forms flexible helical polymers in vitro (Ciuffa et al., 2015). The PB1 domain constitutes the scaffold in p62 filaments, while the LIR and UBA domains are exposed (Ciuffa et al., 2015). We found that the plant ortholog from *Arabidopsis* (AtNBR1) has a PB1 domain that can homopolymerize (Svenning et al., 2011). The presence of a PB1 domain alone is not enough to predict self-interaction. Studies are therefore needed to determine how widespread polymerization is among non-metazoan NBR1 orthologs. Metazoan NBR1 orthologs have lost the basic binding surface resulting in a monomeric PB1 domain. To compensate, metazoan NBR1 and some fungal orthologs harbor a self-interacting CC domain, a domain absent in plant orthologs or p62. Despite the split of the ancestor NBR1 into p62 and NBR1 in metazoans, mammalian NBR1 remains attached to p62 via the acidic PB1 surface that is not mutated (Lamark et al., 2003). The only known interaction partners of mammalian NBR1 that bind via PB1-PB1 interactions are p62 (Lamark et al., 2003) and the kinase MEKK3 (Hernandez et al., 2014), and NBR1 is always recruited to p62 bodies.

We propose that the early metazoan gene duplication facilitated the evolution and divergence in domain structures, which allowed p62 and NBR1 to both tackle separate functions and collaborate on certain functions. The split into two proteins enabled different expression levels in cells and various tissues and different regulations by posttranslational modifications. The gene duplication enabled a deletion of domains from p62 streamlining it as an effective SAR facilitating p62 body formation, which requires high quantities of p62. NBR1 is less central in forming the scaffold of the p62 body, allowing the development of other functions such as gain of the AH domain enabling membrane binding. In humans, NBR1 is much less abundant in most cell types than p62, varying from 10 to almost 100-fold difference in protein levels (Cho et al., 2022; Wang et al., 2015). According to The Human Protein Atlas, both proteins are expressed in most tissues with little tissue specificity, but with particularly high levels of p62 in skeletal muscle and of NBR1 in late spermatids of the testis (Uhlen et al., 2015).

## Plant NBR1 is polymeric, forms filaments similar to p62, and acts in stress responses

*Arabidopsis* NBR1 (AtNBR1) and mammalian p62 share the abilities of PB1 self-polymerization and helical filament formation, as well as LIR-ATG8 binding and UBA-ubiquitin interactions (Jakobi et al., 2020; Svenning et al., 2011). AtNBR1 forms cellular bodies with a striking similarity to those formed by mammalian p62, and the formation of AtNBR1 bodies depends on PB1-mediated polymerization and UBA-mediated ubiquitin binding (Svenning et al., 2011). High-resolution cryo-EM studies of the purified PB1 domain of AtNBR1 revealed similar types of filamentous structures as seen for the human p62 PB1 domain (Jakobi et al., 2020). A tandem arginine motif that is absent in human NBR1, but present in p62 (R21/22) and AtNBR1 (R19/20), is important for stabilizing a filamentous structure and for the formation of p62/AtNBR1 bodies with ubiquitin (Jakobi et al., 2020; Lin et al., 2017). This strongly supports the conclusion that p62 bodies and AtNBR1 bodies are structurally very similar. Another common feature of p62 and AtNbr1 is that their degradation by autophagy depends on a polymeric PB1 domain (Svenning et al., 2011). In comparison, mammalian NBR1 has a monomeric PB1 domain, and its degradation by autophagy does not depend on its PB1 domain (Kirkin et al., 2009).

The roles of NBR1-mediated selective autophagy in plant stress responses have recently been excellently reviewed (Zhang and Chen, 2020; Fig. 1 A). AtNBR1 is involved in heat tolerance, modulation of plant heat memory, plant–pathogen interactions, and aggrephagy (autophagic degradation of protein aggregates) during abiotic stress tolerance (Young et al., 2019; Zhou et al., 2013; Zhou et al., 2014). Upon high-temperature stress, plants require an equilibrium between poststress growth recovery and the establishment of heat stress memory (which relates to heat tolerance complexes being available during the early stages of a high-temperature event; Sedaghatmehr et al., 2019). The HSP90.1-ROF1 complex mediates the heat stress response through interaction with transcription factor HSFA2. A heat-responsive interaction between HSP90.1-ROF1 and HSFA2 in the cytoplasm leads to nuclear translocation and activation of heat-responsive genes. AtNBR1-mediated selective autophagy of HSP90.1 and ROF1 mitigates the HSFA2-dependent response to high temperature (Thirumalaikumar et al., 2021). Consequently, the heat stress response is attenuated. The degradation of heat-responsive elements like HSP90.1 and ROF1 promotes recovery after heat stress but weakens heat stress memory.

Following viral infection, autophagy is often initiated to curtail a viral particle increase by delivering viruses or their components to the lysosomes for degradation, a process known as xenophagy. AtNBR1-dependent selective autophagic degradation of both non-assembled and particle-associated P4 (one of the six cauliflower mosaic virus [CaMV] viral proteins important for viral particle assembly) is ubiquitin-independent and restricts CaMV infection in a process resembling mammalian xenophagy (Hafren et al., 2017). Since particle functions are imperative for successful CaMV infection in plants, AtNBR1-mediated xenophagy counteracts infection establishment. Beyond targeting of non-assembled and particle-associated proteins, RNA silencing is regarded as the main antiviral defense mechanism in plants, and viral suppressors of RNA silencing (VSRs) have co-evolved to escape this mechanism (Boualem et al., 2016). AtNBR1 has been shown to degrade the viral RNA silencing suppressor helper component proteinase (HCpro) of the Turnip mosaic virus (TuMV) by targeting ubiquitinated potyvirus-induced RNA granules (PGs) for autophagic destruction (Hafren et al., 2018; Fig. 1 A).

Unlike viruses, bacteria generally do not enter plant cells due to the plant cell wall and turgor pressure. Instead, bacteria express effector proteins that can be translocated into the plant cells and they manipulate the host cell to promote infection (Khan et al., 2018). NBR1-mediated autophagy has been shown to counteract the pathogenic effect of the bacterial effector protein HopM1, thereby suppressing bacterial proliferation (Ustun et al., 2018). Recently, it was demonstrated that NBR1 directly targets and promotes the selective degradation of the effector protein XopL of the plant bacterium *Xanthomonas campestris pv. Vesicatoria* (Leong et al., 2022). XopL suppresses autophagy through its E3 ligase activity, while also being targeted by NBR1-mediated selective autophagy. Furthermore, NBR1 restricts oomycete *Phytophthora infestans* infection (Dagdas et al., 2018)*.* These studies demonstrate the complexity of host–pathogen interactions and an important role of NBR1 in counteracting infection in plants.

## Aggrephagy—Roles of NBR1 in p62 bodies

**Figure 4.**
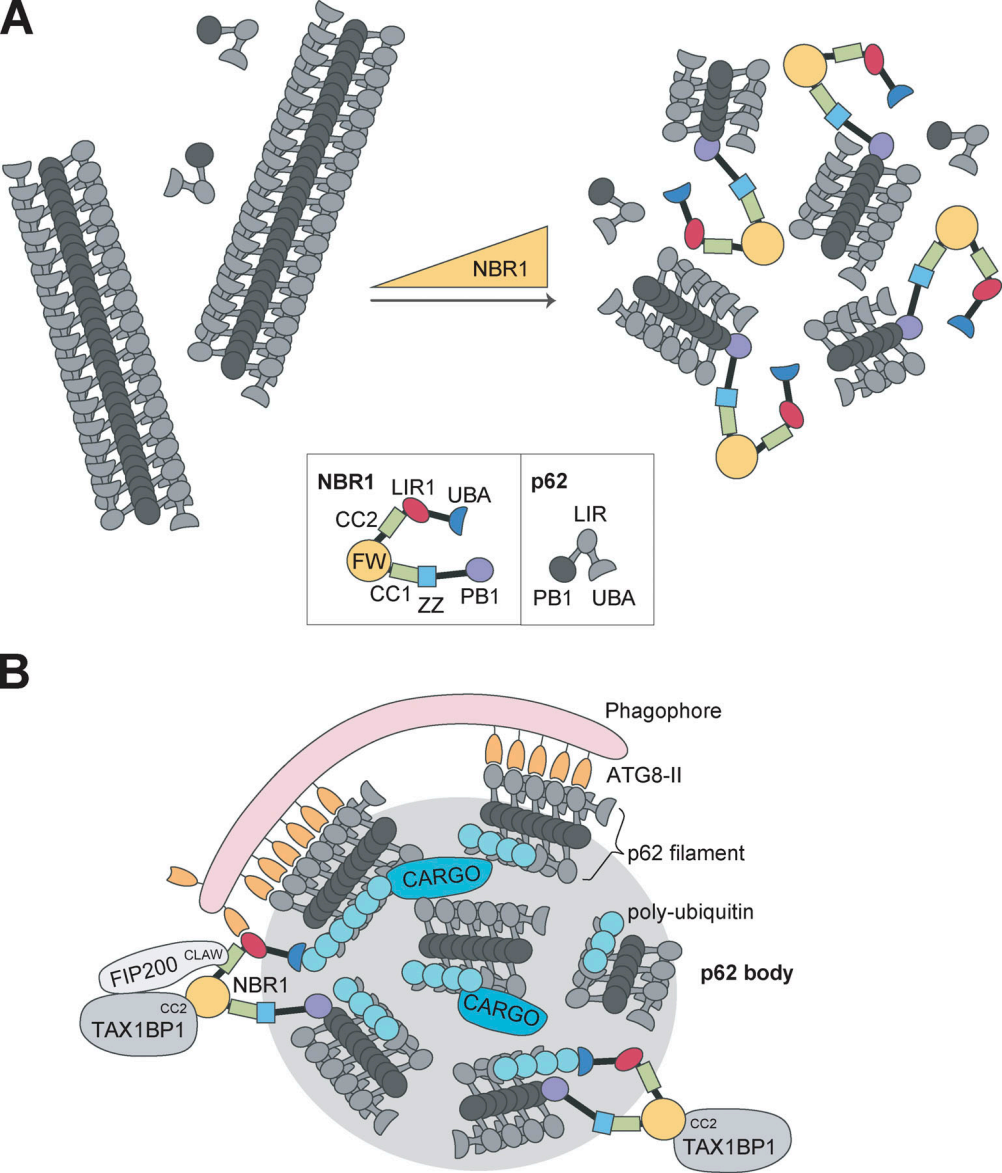
**NBR1 collaborates with p62 in the formation of p62 bodies and with TAX1BP1 in the recruitment of core autophagy components to p62 bodies. (A)** p62 forms long filaments in vitro as a result of PB1-mediated polymerization. Due to the monomeric nature of NBR1, it is hypothesized that NBR1 can act as a chain terminator of p62 filaments. With increasing amounts of NBR1 in vitro, the length of the p62 filaments is reduced. Shorter p62 filaments will likely form p62 bodies more easily. Therefore, a role for NBR1 in cells may be to promote p62 body formation by regulating p62 filament length. **(B)** The role for NBR1 in p62 body dynamics. NBR1 promotes p62 body formation by PB1-mediated regulation of p62 filament length and high-affinity ubiquitin binding. Furthermore, NBR1 facilitates autophagosome formation by recruiting TAX1BP1 and FIP200. FIP200 is recruited by direct binding between the FIP200 CLAW domain to the CC2 of NBR1. TAX1BP1 binds NBR1 FW domain via its CC2 domain and also recruits FIP200. NBR1 LIR1 also binds ATG8 proteins in the growing phagophore. In addition, NBR1 contains multiple domains that may be involved in cargo recruitment (UBA, ZZ, FW, AH).

Depletion of NBR1 inhibits the formation of p62 bodies (Kirkin et al., 2009). Human NBR1 binds to p62 by strong PB1–PB1 electrostatic interactions and competes with p62 polymerization, acting as a chain terminator. Hence, in vitro, the addition of NBR1 reduces filament length (Jakobi et al., 2020). The role of NBR1 may therefore be to regulate the length of p62 filaments in p62 bodies. By reducing filament length, NBR1 may promote the formation of p62 bodies since very long filaments are not easily packed into dynamic, phase-separated structures (Fig. 4 A). The addition of purified NBR1 increases in vitro phase separation of p62 upon mixing with ubiquitin (Zaffagnini et al., 2018). In mouse hepatocytes, the formation of p62 bodies is compromised by the loss of NBR1 and promoted by overexpression of NBR1 (Sanchez-Martin et al., 2020).

Using a combination of in vitro reconstitution assays and cell biological studies, NBR1 contributes to efficient cargo clustering in p62 bodies by bringing its high-affinity ubiquitin-binding UBA domain to the p62 filaments via PB1–PB1 interactions between NBR1 and p62 (Turco et al., 2021). NBR1 uses its FW domain to recruit TAX1BP1 to p62 filaments, and the core autophagy machinery component FIP200 of the ULK complex is recruited by both TAX1BP1 and by NBR1. The SKICH domain of TAX1BP1 (and NDP52) is known to bind to FIP200 (Ravenhill et al., 2019), while NBR1 binds to FIP200 via its CC2 domain (Turco et al., 2021; Fig. 4 B). Previously, it was shown that p62 bound to the C-terminal Claw domain of FIP200 (Turco et al., 2019). NBR1 also binds to the Claw domain, but much more strongly than p62, even somewhat stronger than TAX1BP1. However, TAX1BP1 is suggested to be the main recruiter of FIP200 to p62 bodies. The Claw domain in FIP200 is homologous to the C-terminal region of the yeast selective autophagy adaptor and Atg1 activator, Atg11 (Turco et al., 2019). The yeast NBR1 homolog, Atg19, recruits Atg11 by binding to this C-terminal region in Atg11 (Yorimitsu and Klionsky, 2005). Hence, the parallel here is clear between mammalian NBR1 and FIP200 and yeast Atg19 and Atg11. In contrast to p62 and NBR1, TAX1BP1 does not contribute directly to the formation of ubiquitin condensates in vitro (Turco et al., 2021) or in cells treated with puromycin that causes ubiquitinated protein aggregates to form in cells (Sarraf et al., 2020). However, TAX1BP1 is needed for the efficient autophagic degradation of these aggregates, and mice expressing a deletion mutant of TAX1BP1 that cannot bind ubiquitin show the accumulation of ubiquitin-conjugated proteins and Lipofuscin pathology (Sarraf et al., 2020). Upon ATG7-independent autophagy in K562 cells, NBR1 forms a heterotypic autophagy receptor complex with p62 and TAX1BP1 that requires TAX1BP1 to induce local autophagosome formation (Ohnstad et al., 2020). TAX1BP1 binds to NBR1 via its CC2 domain. Taken together, these studies show that in human cells, a trio of SLRs work together for the efficient formation and degradation of p62 bodies. NBR1 affects p62 filament length by PB1 domain interactions, as well as recruitment of ubiquitinated cargo via the UBA domain, recruitment of TAX1BP1 via the FW domain, FIP200 via the CC2 domain, and ATG8s via the LIR1 domain (Fig. 4).

Phase separation of plant NBR1 has not been demonstrated experimentally. However, AtNBR1 ectopically expressed in HeLa cells or plant tissues form ubiquitin aggregates resembling those formed by p62 (Svenning et al., 2011). We therefore believe that AtNBR1/p62 bodies represent a unique type of preautophagic structures or phagophore assembly sites (PAS) that are evolutionary conserved and formed in all eukaryotic cells expressing p62 or polymeric NBR1 orthologs.

A functionally distinct type of p62 bodies named dendritic aggresome-like induced structures (DALIS) are transiently formed by p62 in activated dendritic cells and involved in antigen processing (Lelouard et al., 2004; Lelouard et al., 2002). Ubiquitinated substrates recruited to DALIS, including defective ribosomal products (DRiPs), are either degraded by the proteasome or by autophagy. NBR1 is not required for the formation of DALIS, but for their degradation by autophagy and antigen presentation via MHC class II, which may occur even in the absence of p62 (Arguello et al., 2016). In cells lacking NBR1, ubiquitinated substrates in DALIS are degraded by the proteasome, presumably depending on the solubilization of DALIS. Puromycin induction of p62 bodies in HeLa cells involving recruitment of ubiquitinated DRiPs into p62 bodies is highly dependent on NBR1 (Kirkin et al., 2009). While the degradation of p62 does not depend on NBR1, ubiquitinated proteins in p62 bodies are not degraded by autophagy in cells lacking NBR1 (Kirkin et al., 2009).

NBR1 is efficiently degraded by ATG7- and ATG8-dependent autophagy independent of p62 (Kirkin et al., 2009), but also ATG7-independent autophagy pathways exist for NBR1. The SLRs p62, NBR1, TAX1BP1, and NDP52 are degraded by endosomal microautophagy in response to acute starvation (Mejlvang et al., 2018). Degradation of NBR1 is in this case partially ATG7-independent. ATG7-independent degradation of NBR1 and TAX1BP1 requires direct interaction of NBR1 with TAX1BP1 (Ohnstad et al., 2020) and also depends on a SKICH-mediated binding of TAX1BP1 to FIP200 (Ravenhill et al., 2019; Thurston et al., 2016). Hence, the SLR-dependent recruitment of FIP200 allows ATG8-independent autophagy to occur to degrade NBR1 in the absence of functional conjugation machinery mediating lipidation of ATG8s (Ohnstad et al., 2020).

## Pexophagy

NBR1 acts as a receptor for pexophagy in mammalian cells (Deosaran et al., 2013; Fig. 1 A). Interestingly, the NBR1 homolog in the filamentous ascomycete *Sordaria macrospora* is required for pexophagy, and human NBR1 can rescue growth defects under stress conditions when the fungal protein is lost (Werner et al., 2019). In *Arabidopsis*, pexophagy occurs independently of AtNBR1 (Young et al., 2019). We found that the amphipathic alpha-helix (AH) located immediately N-terminal to the UBA domain as well as the UBA, LIR, and CC domains of mammalian NBR1 are required for pexophagy. Coincident binding of the AH and UBA domains directs NBR1 to ubiquitinated peroxisomes and targets them for selective autophagy. Electron microscopy studies revealed that aggregates of overexpressed NBR1 contain clusters of 50-nm vesicles together with peroxisomes, autophagosomes, and some larger vesicle structures (possibly late endosomes; Deosaran et al., 2013). Endogenous p62 is recruited to NBR1 vesicle aggregates via its direct binding to NBR1. Its presence has a positive effect on NBR1-mediated pexophagy. However, pexophagy occurs also in the absence of p62, and p62 overexpression does not induce pexophagy (Deosaran et al., 2013).

Activation of the hypoxia-inducible factor HIF-2α augments NBR1-mediated pexophagy, and peroxisome numbers are reduced in VHL-deficient human clear cell renal cell carcinomas with elevated levels of HIF-2α (Walter et al., 2014). Overexpression of the peroxisomal membrane protein PEX3 increases NBR1-mediated pexophagy, but it is not ubiquitination of PEX3 as such that leads to increased pexophagy (Yamashita et al., 2014). SQSTM1/p62 was required only for the clustering of peroxisomes. The peroxisomal E3 ubiquitin ligase peroxin 2 (PEX2) is upregulated upon amino acid starvation and rapamycin treatment. PEX2 expression induces ubiquitination of PEX5 and PMP70 on peroxisomes and boosts NBR1-dependent pexophagy (Sargent et al., 2016).

## Xenophagy

Several soluble SLRs are involved in the autophagic clearance of invading pathogens, a process known as xenophagy (Goodall et al., 2022; Lamark and Johansen, 2021). Invading bacteria exposed in the cytosol become tagged with ubiquitin chains and sequestered into autophagosomes by ubiquitin-binding SLRs (Goodall et al., 2022). NBR1, NDP52, and p62 are recruited to intracytosolic *Mycobacterium tuberculosis* (Manzanillo et al., 2013) and *Shigella flexneri* (Mostowy et al., 2011; Fig. 1 A). In the case of *S. flexneri* infection, NBR1 is necessary to recruit p62 and NDP52; yet the mechanism and functional significance of this remains elusive (Mostowy et al., 2011). Upon infection with *M. tuberculosis*, both NBR1 and p62 are recruited in a parkin2-dependent manner (Manzanillo et al., 2013). NBR1, but not p62, can also be recruited by the HECT E3 ligase Smurf1 to *M. tuberculosis* (Franco et al., 2017). Mice depleted of either parkin2 or Smurf1 are more sensitive to *M. tuberculosis* infection, suggesting that both E3 ligases are important for the recruitment of SLRs and subsequent xenophagy (Franco et al., 2017; Manzanillo et al., 2013). Furthermore, the *M. tuberculosis* surface protein Rv1468c binds polyubiquitin chains and recruits SLRs, including NBR1, in a UBA-dependent manner (Chai et al., 2019). NBR1 is also recruited to group A Streptococcus (GAS)-containing vesicles in a Tollip-dependent manner. Tollip knockout prevents the recruitment of NBR1, NDP52, and TAX1BP1 to GAS-containing vesicles, yet p62 recruitment is unaffected (Lin et al., 2020).

Viruses utilize various strategies to manipulate host cell autophagy to their advantage (Liu et al., 2022). One such strategy involves NBR1-mediated autophagic degradation of the anti-viral adaptor protein MAVS (Zeng et al., 2021). The basic polymerase 1 (PB1, not to be confused with the PB1 domain) of the H7N9 strain of influenza A virus promotes K27-polyubiquitination of MAVS and specifically recruits NBR1 to mediate enhanced MAVS degradation by autophagy, which further facilitates viral replication. Interestingly, the autophagic degradation of MAVS is dependent on ATG7, but does not require components of the ULK-complex. Some viruses, like Coxsackievirus, also counter the antiviral activity of both p62 and NBR1 by encoding proteases that cleave p62 and NBR1, releasing C-terminal fragments exerting dominant negative effects on endogenous p62 and NBR1 (Shi et al., 2014).

## NBR1 and human disease

### Proteinopathies

The giant protein titin acts as a scaffold for the assembly of the sarcomere and signaling complexes in muscle cells. Titin contains a serine/threonine kinase domain (TK) involved in mechanosensing (Puchner et al., 2008). NBR1 interacts with TK and recruits p62 and the E3 ligase MURF2 in active muscle, thereby regulating mechanical signaling and muscle gene transcription (Lange et al., 2005). Analysis of two unrelated families with hereditary myopathy with early respiratory failure identified a mutation in the NBR1-interaction site within TK that disrupts NBR1 binding. NBR1 was more diffusely localized, p62 accumulated in many patient muscle samples, and MURF2 showed more nuclear localization, suggesting disruption of the NBR1–p62–MURF2 complex.

NBR1 function in aggrephagy may link it to diseases characterized by the accumulation of misfolded proteins. One such disease is sporadic inclusion body myostitis (sIBM), a progressive degenerative myopathy that is the most common skeletal myopathy in older people. Pathological features of this disease include the accumulation of rimmed vacuoles (hence the name “inclusion body”) and misfolded protein aggregates in muscle fiber cells, indicating defects in autophagy and lysosomal degradation. Biopsies and cultured cells from sIBM patients show an increase in NBR1 protein, and NBR1 accumulation alongside p62 and LC3 in the ubiquitin-positive aggregates that are characteristic of this disease (D’Agostino et al., 2011). NBR1 is phosphorylated by GSK3B at Thr586, which promotes NBR1-mediated degradation of ubiquitinated proteins and prevents the formation of misfolded aggregates (Nicot et al., 2014). Meanwhile, in sIBM patient biopsies, NBR1 phosphorylation is reduced. This, in turn, prevents the clearance of ubiquitinated substrates, instead leading to the accumulation of misfolded proteins. NBR1 is also accumulated in Lewy bodies in Parkinson’s disease and glial cytoplasmic inclusions in multiple system atrophy (Odagiri et al., 2012). We also reported early on that NBR1 colocalized with p62 and ubiquitin in Mallory bodies in the liver of a patient with alcoholic steatohepatitis (Kirkin et al., 2009). However, given the cooperation of NBR1 and p62 in p62 bodies, it is often hard to distinguish NBR1-specific pathological effects.

### NBR1 and cancer

Data from the Human Protein Atlas show that NBR1 mRNA is expressed in most cancers with low-cancer specificity (Uhlen et al., 2017). Recently, whole exome sequencing on germline DNA from a family presenting with different subtypes of renal cell carcinoma (RCC) identified a frameshift mutation in NBR1 (Adolphe et al., 2021). The mutation results in the expression of a truncated form of NBR1 that no longer includes LIR and UBA domains. While this does not affect the ability of NBR1 to interact with itself and with p62, overexpression of the truncated NBR1 delays the turnover of p62 and peroxisomes. The overexpression of truncated NBR1 increases the proliferation capacity of renal cancer cells compared with cells overexpressing WT NBR1. Exactly how NBR1 may affect RCC development is unclear and will require further studies. Some rare cases of RCC present with eosinophilic cytoplasmic inclusions, which are aggregates associated with membrane-bound, electron-dense organelles (Yu et al., 2018). These aggregates contain p62, NBR1, and other autophagy markers, and maybe the result of defects in autophagy. Strikingly, these aggregates are surrounded by clusters of peroxisomes only when NBR1 is present. While these inclusions are relatively rare in RCC, their presence is generally associated with larger tumors (Yu et al., 2018). However, the exact effects of these inclusions and clustering of peroxisomes on tumor progression are not known.

In recent years, several comprehensive studies have revealed potentially unique roles for NBR1 in cancer development, independent of p62. In migrating cells, focal adhesions (FAs) are continuously assembled and disassembled to allow cell protrusion, adhesion, and contraction. NBR1 has a specific role as a selective autophagy receptor in the turnover of FAs at the leading edge of the cell during cell migration (Kenific et al., 2016; Fig. 1 A). Knockdown of NBR1, but not other SLRs, inhibited cell migration and increased FA lifetime. NBR1 localizes to FAs and recruits autophagosomes to FAs at the leading edge of the cell, targeting FAs for autophagosomal degradation and thereby promoting cell migration. This process may be hijacked by cancer cells to facilitate cancer metastasis. A more recent study investigated the role of autophagy in different stages of breast cancer development in a mouse model (Marsh et al., 2020). As expected, impairment of autophagy led to an accumulation of NBR1 and p62. Intriguingly, the accumulation of NBR1, but not p62, promoted the development of an aggressive subpopulation of tumor cells. Injection of tumor cells overexpressing NBR1 led to metastatic outgrowth, while overexpression of p62 did not. Knockdown of NBR1 in the autophagy-deficient cells reversed the metastatic phenotype otherwise observed upon autophagy inhibition alone. Even in autophagy-competent cells, the ectopic overexpression of NBR1 was sufficient to promote tumor metastasis. These results suggest that aberrant accumulation of NBR1 can promote metastatic outgrowth during breast cancer progression.

NBR1 plays a role in the immune evasion of pancreatic cancer cells. Cytotoxic T cells can detect and eliminate cancerous cells that present tumor antigens via MHC class I molecules on their surface. Consequently, many cancers evade the immune system through mutations or loss of MHC class I molecules. In pancreatic ductal adenocarcinomas (PDACs), MHC class I surface expression is often downregulated, but rarely due to mutations. In PDAC cells, MHC class I molecules are being degraded by NBR1-mediated selective autophagy, effectively preventing them from reaching the cell surface (Yamamoto et al., 2020; Fig. 1 A). Inhibition of autophagy increases both the total and cell surface expression of MHC class I in PDAC cells and further leads to increased antigen presentation, T-cell infiltration, and tumor cell killing. Of the SLRs tested, NBR1 was found to co-precipitate with MHC class I proteins, while p62, NDP52, TAX1BP1, and OPTN did not co-precipitate. Knockdown of NBR1 increases the total and surface levels of MHC class I molecules. Altogether, this supports a role for NBR1 in targeting MHC class I molecules for autophagic degradation, facilitating immune evasion of PDAC cells. More studies are required to probe for possible direct or indirect roles of NBR1 and other SLRs on the turnover of MHC class I in different normal and cancer cells.

## Possible autophagy-independent roles of NBR1

NBR1 has been implicated in processes with no obvious link to autophagy, including the downregulation of receptor tyrosine kinases and inhibition of ERK1/2 (Mardakheh et al., 2010; Mardakheh et al., 2009). Several PB1 domain-containing proteins are implicated in the differentiation of activated T cells, including NBR1 and p62 (Martin et al., 2006; Yang et al., 2010). NBR1 is required for proper differentiation of T helper 2 cells. Whether this relates to the function of NBR1 as an autophagy receptor or potentially autophagy-independent functions is not known. NBR1 also acts as a regulator of JNK signaling and adipose tissue inflammation by engaging in a PB1–PB1 interaction with MEKK3 (Hernandez et al., 2014). Furthermore, p62 and NBR1 regulate PPARγ–RXRα heterodimerization to control thermogenesis in brown adipocytes. NBR1 represses the activity of PPARγ when p62 is inactivated. (Huang et al., 2021). NBR1 has been shown to deliver IL-12 to late endosomes in intestinal myeloid cells (Merkley et al., 2022).

NBR1 is reported to interact with activated p38 and limit its activity (Kim et al., 2019; Whitehouse et al., 2010). In mice, the expression of a truncated version of NBR1 that is unable to bind p38 and mitigate its activity results in an age-dependent increase in bone mass (Whitehouse et al., 2010). Furthermore, loss of NBR1 in both transformed and non-transformed cell lines causes cellular senescence because of p38-induced ER stress (Kim et al., 2019). Whether or not this negative regulation of p38 requires autophagy is not clear.

## Concluding remarks and future questions/perspectives

NBR1 is the archetypal autophagy receptor, likely present as early as in the latest eukaryotic common ancestor. Gene duplication in the early metazoan lineage and subsequent molecular evolution gave rise to mammalian NBR1 and the much more studied paralog p62. Studies of NBR1 homologs in plants, fungi, and mammals are beginning to shed light on some of the unique roles of NBR1, gradually bringing it out of the shadow of p62. Structural and functional comparisons clearly suggest that yeast Atg19 and Atg34 are NBR1 homologs. NBR1 plays a central role in pexophagy in mammals, while its role in xenophagy is far better understood in plants than in mammals. Exciting new information has come from studies on the collaboration between p62, TAX1BP1, and NBR1 in the recruitment of core autophagy components to p62 bodies to facilitate autophagosome formation. Here, NBR1 plays a much more central role than anticipated. Important p62-independent roles of NBR1 in cancer metastasis and immune evasion in cancer have been revealed. NBR1 also has autophagy-independent roles in regulating signaling pathways and immune cell differentiation.

NBR1 is understudied and future research must address this. In future studies, a deeper understanding of the evolution and interplay between NBR1 and p62 may reveal further functions of NBR1, not only as a SAR but also in regulating the dynamics of p62 bodies. Very likely, new autophagic substrates unique to NBR1 will be discovered. It will be important to penetrate more mechanisms of pexophagy and xenophagy involving NBR1. Studies of fungal NBR1 homologs ask the question if there are analogous roles of mammalian NBR1 in pathways similar to the Cvt and NVT pathways. Further elucidation of pathophysiological roles of NBR1 in human disease is required to evaluate NBR1 as a potential target for therapeutic strategies in cancer and proteinopathies.
